# Panel testing reveals nonsense and missense *CDH1* mutations in families without hereditary diffuse gastric cancer

**DOI:** 10.1002/mgg3.197

**Published:** 2016-01-13

**Authors:** Julie M. Huynh, Christina M. Laukaitis

**Affiliations:** ^1^ Department of Molecular and Cellular BiologyUniversity of ArizonaTucsonArizona; ^2^ Department of MedicineCenter for Applied Genetics and Genomics and University of Arizona Cancer CenterCollege of MedicineUniversity of ArizonaTucsonArizona

**Keywords:** Ataxia telangiectasia mutated, breast cancer risk, E‐cadherin, germline mutation, hereditary diffuse gastric cancer

## Abstract

**Background:**

The reported penetrance of germline *CDH1* mutations is high in families with hereditary diffuse gastric cancer (HDGC). Men and women have a 70% and 56%, respectively, cumulative risk of developing diffuse gastric cancer by age 80. Women additionally have a 42% cumulative risk of developing breast cancer. Due to the high penetrance of these mutations, prophylactic total gastrectomy is currently recommended for *CDH1* mutation carriers. However, whether everyone with a *CDH1* gene mutation is at risk for HDGC is not clear.

**Methods:**

Mutation identification was performed by next‐generation sequencing. Mutations and variant status was confirmed by Sanger sequencing in 11 family members.

**Results:**

We present two families with pathogenic *CDH1* mutations. The first family carries a novel truncating, nonsense *CDH1* mutation that we were able to trace for three generations, but reports no family history of diffuse gastric cancer. The occurrence of cancer in this family deviates significantly from the expectation for HDGC. The proband from the second family presents with breast cancer and carries a previously reported pathogenic *CDH1* mutation, but also reports no family history of diffuse gastric cancer.

**Conclusions:**

Our study demonstrates the need for further analysis of *CDH1* mutation penetrance in order to better counsel asymptomatic *CDH1* mutation carriers on preventative measures and general care.

## Introduction

Germline mutations in the *E‐cadherin* (*CDH1*; OMIM #192090) gene are associated with the autosomal dominant cancer susceptibility syndrome, hereditary diffuse gastric cancer (HDGC) (Guilford et al. 1998). *CDH1* mutations have been found in greater than 50% of families with HDGC (Kaurah et al. 2007). In families that met clinical criteria for HDGC, men carrying pathogenic *CDH1* mutations were found to have a 70% cumulative risk of developing gastric cancer by age 80, while women have a 56% cumulative risk of developing gastric cancer by age 80 (Hansford et al. 2015). In addition to the risk of diffuse gastric cancer development, women carrying *CDH1* mutations have a 42% cumulative risk of developing breast cancer (typically lobular breast cancer) (Hansford et al. 2015).

The *CDH1* gene maps to chromosome 16q22.1 and encodes an epithelial cadherin protein, E‐cadherin (Nagar et al. 1996; Natt et al. 1989). E‐cadherin is part of the cadherin superfamily of transmembrane glycoproteins that are involved in calcium dependent cell–cell adhesion and invasion suppression (Berx et al. 1998). Cells gain invasive properties when E‐cadherin is mutated due to the loss of cell–cell adhesion (Perl et al. 1998).

Because of the link between E‐cadherin and cancer, *CDH1* mutations are assessed in various cancer risk gene panels. Extended cancer risk panels typically include multiple genes with a variety of risks conferred for a variety of cancer types. *Ataxia Telangiectasia Mutated* (*ATM*; OMIM #607585) is another gene that is assessed in some of these panels. *ATM* maps to chromosome 11q22‐q23 (Gatti et al. 1988). It encodes ATM protein, a serine/threonine protein kinase involved in DNA damage response (Lavin 2008). Germline mutations in *ATM* are associated with an increased risk for breast cancer and pancreatic cancer (Roberts et al. 2012).

The introduction of extended cancer risk gene panels have allowed for screening of many cancer risk genes simultaneously. Thus, mutations in genes that would be unexpected given the clinical presentation and family history of patients are inevitably found. Indeed, studies are emerging where patients with breast cancer and who have a family history of breast cancer are found to carry a *CDH1* germline mutation, though they do not have a family history of gastric cancer (Lajus and Sales 2015; Schrader et al. 2008; Xie et al. 2011). However, prophylactic total gastrectomy is currently recommended for *CDH1* mutation carriers due to reported high penetrance (van der Post et al. 2015).

Here, we describe an asymptomatic proband who was found to carry a novel truncating mutation in *CDH1* and a missense variant of unknown significance in *ATM*. She has no family history of gastric cancer and limited family history of breast cancer. We were able to trace the *CDH1* mutation for three generations and the *ATM* variant of unknown significance for two generations. All tested individuals, including the proband, her mother, and her grandfather are currently healthy and have never had cancer. We additionally describe the proband of a second family who was found to carry a germline *CDH1* mutation disrupting a splice site. She has had lobular and ductal breast cancer, but no personal or family history of diffuse gastric cancer. Clearly, further characterization of the phenotype and cancer risks of *CDH1* mutation carriers will be needed in order to guide screening and surgical interventions appropriately.

## Materials and Methods

### Mutation identification

Next‐generation sequencing was performed by commercial laboratories to identify mutations in a panel of breast cancer‐related genes. Mutations or variants of unknown significance were identified in *CDH1* (NM_004360) and *ATM* (NM_000051). Sanger sequencing was performed by a commercial laboratory in 11 family members to confirm mutation and variant status.

### Statistical analysis

We conservatively estimated the number of people expected to have developed gastric cancer in a family of six members with the age distribution of Family 1 using published penetrance estimates (Pharoah et al. 2001). Family 1 has one female member aged 39 years (counted in cumulative risk by age 30 category with a reported 4% risk of gastric cancer), one aged 44 (in risk to age 40 category with 21% penetrance), one aged 64 (risk to 60 with 64% penetrance), and one aged 70 (risk to 70 with 71% penetrance). Males are aged 47 and 93 (9% and 67% penetrance, respectively). We summed these risks (0.04 + 0.21 + 0.64 + 0.71 + 0.09 + 0.67) to estimate that 2.36 people from a family of six members with this age distribution should have developed gastric cancer. We compared expected proportion (2.36/6 = 0.3633) to zero (number observed with cancer) using an exact binomial test.

## Results

The Family 1 proband is a 44‐year‐old female of European ancestry who was seen in our high‐risk cancer genetics clinic for interpretation of results of genetic testing. An extended cancer risk panel was ordered by her gynecologist during her yearly wellness visit because of a history of cancer in the extended family. The results revealed that she is heterozygous for a pathogenic mutation in *CDH1* and heterozygous for a variant of unknown significance in *ATM*. The patient showed no weight loss, abdominal pain, breast lumps, dyspepsia, or history of abnormal mammogram or breast skin changes.

The truncating *CDH1* mutation the proband carries is a G to T substitution at nucleotide position 172 (c.172G>T). This mutation has not been previously reported in the literature. It is located in coding exon 3 (of 16 coding exons) and results in a change from the amino acid glutamic acid at position 58 to a stop codon (p.E58*), ultimately resulting in the loss of exons 4–16.

Similar truncating mutations have been reported in families with HDGC. One HDGC family with two confirmed cases of gastric cancer and two unconfirmed cases of gastric cancer carries a C to T substitution at nucleotide position 187 (c.187 C>T; p.R63*) in *CDH1* (Gayther et al. 1998). Like the *CDH1* mutation found in the proband and her family, this mutation is located in coding exon 3. Another family with an extensive unconfirmed history of diffuse gastric cancer (two confirmed cases) carries a p.Q64* mutation (Guilford et al. 1999).

**Figure 1:**
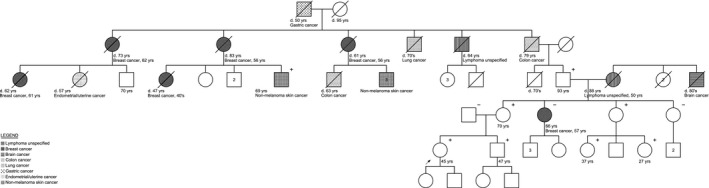
Pedigree of family 1. Arrow depicts the proband. Women are represented by circles. Men are represented by squares. A diagonal slash indicates a deceased family member. (+) indicates family members with the *CDH1* c.172G>T mutation. (−) indicates tested noncarriers. The legend illustrates cancers affecting the family.

To determine whether this mutation in the Family 1 proband was de novo or hereditary, other family members were tested. The following family members who are all currently healthy with no cancer were positive for the *CDH1* c.172G>T mutation: the proband's mother (70‐years‐old), brother (47‐years‐old), maternal aunt (57‐years‐old), and two daughters (aged 29 and 39‐years‐old), and maternal grandfather (93‐years‐old) (Fig 1). One maternal aunt diagnosed with ductal breast cancer at age 57 has tested negative for this mutation.

We compared this low rate of cancer in this family with the expected number of gastric cancers in HDGC families (Pharoah et al. 2001). Finding no cancer in seven family members between the ages of 27 and 93 deviates significantly (*P* < 0.05) from the 2.36 expected in a family with this age distribution.

The only case of gastric cancer in the proband's family occurred in her maternal great‐great‐grandfather, who passed away in 1920 at 50 years of age. We do not have a copy of the original pathological report to confirm this case as gastric cancer. All five of his children died of cancer: two of breast cancer (unknown subtype), one of lymphoma, one of lung cancer, and one of rectal cancer. The proband's family speculates that the large amounts of cancer could possibly be due to the family being downwind of nuclear testing in the 1940s and 1950s. The familial CDH1 mutation has been identified in individual III‐7, a 69‐year old male with squamous cell skin cancer whose sister and mother both died of breast cancer.

The variant of unknown significance in *ATM* the proband carries is a C to A substitution at nucleotide 4964. This results in the replacement of a serine at codon 1655 with a tyrosine. These two amino acids have dissimilar properties, though it is not clear what the effect of this mutation would be on the ATM protein (Data S1). The mutation was also found in the proband's mother, but not in the proband's brother or grandfather (Data S2). We speculate that it came through the proband's maternal grandmother and may explain her diagnosis of Non‐Hodgkins lymphoma at age 50 and the proband's maternal great uncle who died in his 80's of a brain cancer with unknown pathology. The family reports no cancer on her father's side and he tested negative for both mutations (Fig. 1).

**Figure 2:**
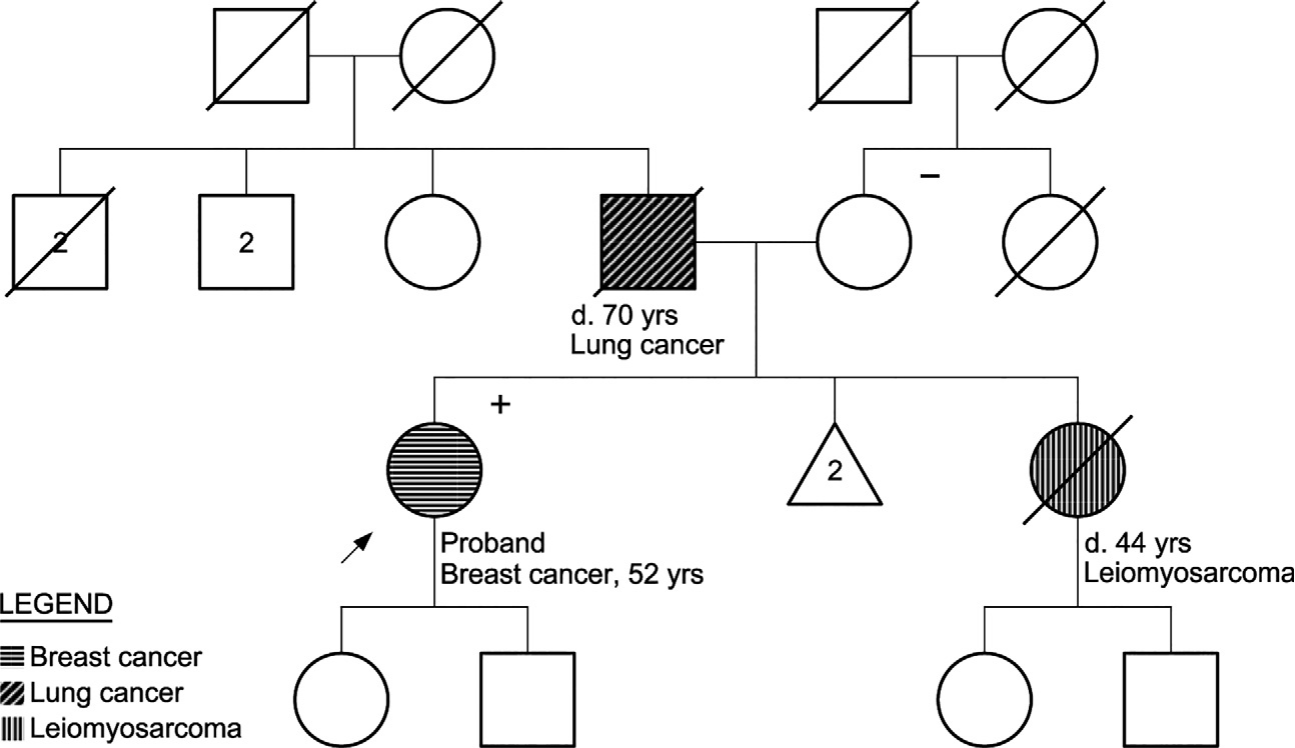
Pedigree of family 2. Arrow depicts the proband. Women are represented by circles. Men are represented by squares. A diagonal slash indicates a deceased family member. (+) indicates family members with the *CDH1* c.1565+1G>A mutation. (−) indicates tested noncarriers. The legend illustrates cancers affecting the family.

At the same time this family was undergoing genetic testing, we saw another patient with a pathogenic *CDH1* mutation. The European ancestry proband was diagnosed with both ductal and lobular breast cancer at 52‐years‐old. A 5‐gene breast cancer panel including *BRCA1, BRCA2*, and *TP53* was ordered because of a family history of a sister with duodenal leimyosarcoma at age 44 and a father with lung cancer at age 70 (Fig. 2). Molecular genetic evaluation revealed the unexpected presence of a pathogenic *CDH1* mutation that substitutes a G to an A in the first nucleotide of intron 10 (c.1565 + 1G>A); this mutation disrupts the splice donor site. This mutation has been previously reported in a family with predominantly lobular breast cancer cases and one early onset gastric cancer case (Schrader et al. 2008). The proband's mother underwent genetic evaluation and does not carry the mutation. Since the proband's father is deceased, we cannot determine whether the proband's mutation is *de novo* or inherited. While it seems feasible that her sister's early cancer could have been related, we find no reports of leiomyosarcoma in *CDH1* mutation carriers and pathology is not available for re‐review. This family's presentation is again complicated by radiation exposure.

## Discussion

The International Gastric Cancer Linkage Consortium has set criteria for characterizing HDGC: two gastric cancer cases (with one individual with confirmed diffuse gastric cancer at any age) OR three confirmed individuals with gastric cancer in first or second degree relatives regardless of age OR a single case of gastric cancer before 40 or personal or family history of gastric cancer and lobular breast cancer, one diagnosed before 50 (van der Post et al. 2015). Neither of the two families presented here meets any of these criteria, yet they are carriers of pathogenic *CDH1* germline mutations.

Because mutations in *CDH1* have been reported as highly penetrant, preventative measures including prophylactic total gastrectomy is currently recommended for carriers (van der Post et al. 2015). The penetrance of gastric cancer in people with *CDH1* mutations has been estimated to range from 83% to 40%, depending on gender and ethnicity (Guilford et al. 1999; Kaurah et al. 2007; Pharoah et al. 2001). Evaluations of *CDH1* mutation penetrance in large families from later studies are lower (Kaurah et al. 2007), suggesting that family ascertainment for HDGC might have skewed early estimates. Penetrance is also lower in families of European ancestry (Guilford et al. 1999). However, the increased availability of extended cancer risk panels might result in more pathogenic *CDH1* germline mutations being found in patients without a family history of HDGC, as is the case with the two families presented here. The vast majority of families with truncating *CDH1* mutations have a history of HDGC; however, at least one other family has been reported to only have a family history of invasive lobular breast cancer, but not HDGC (Xie et al. 2011). It is unclear whether this phenotype reflects a new genotype/phenotype correlation or just an expansion of the general truncating *CDH1* mutation phenotype. Additionally, with the increased usage of these extended cancer risk panels, other cancer risks, such as risks for leiomyosarcoma, that were not previously associated with mutations in *CDH1* might come to light.

Whether there are synergistic effects of radiation exposure and/or second mutations with the *CDH1* mutations are unclear. Further work needs to be done to determine the penetrance of pathogenic *CDH1* mutations and the environmental and/or additional genetic factors that affect the biology.

Advising an invasive procedure with high morbidity and mortality to a family with little history of the disease is uncomfortable, but allowing potential risk to go untreated is even more so. *CDH1* mutation penetrance needs further assessment in order to better advise asymptomatic families with pathogenic mutations regarding preventative measures.

## Conflict of Interest

The authors report no competing interests.

## Supporting information


**Data S1.** Report supporting VUS status of ATM p.S1655Y/c.4964C>A.

**Data S2.** Pedigree of family 1. Arrow depicts the proband. Women are represented by circles. Men are represented by squares. A diagonal slash indicates a deceased family member. (+) indicates family members with the *ATM* p.S1655Y/c.4964C>A variant of unknown significance. (−) indicates tested noncarriers. The legend illustrates cancers affecting the family.
