# Supplementary figures and images for: Panel testing reveals nonsense and missense CDH1 mutations in families without hereditary diffuse gastric cancer
Source: Mol Genet Genomic Med. 2016 Jan 13;4(2):232–6. doi: 10.1002/mgg3.197 (PMC4799867; doi:10.1002/mgg3.197)

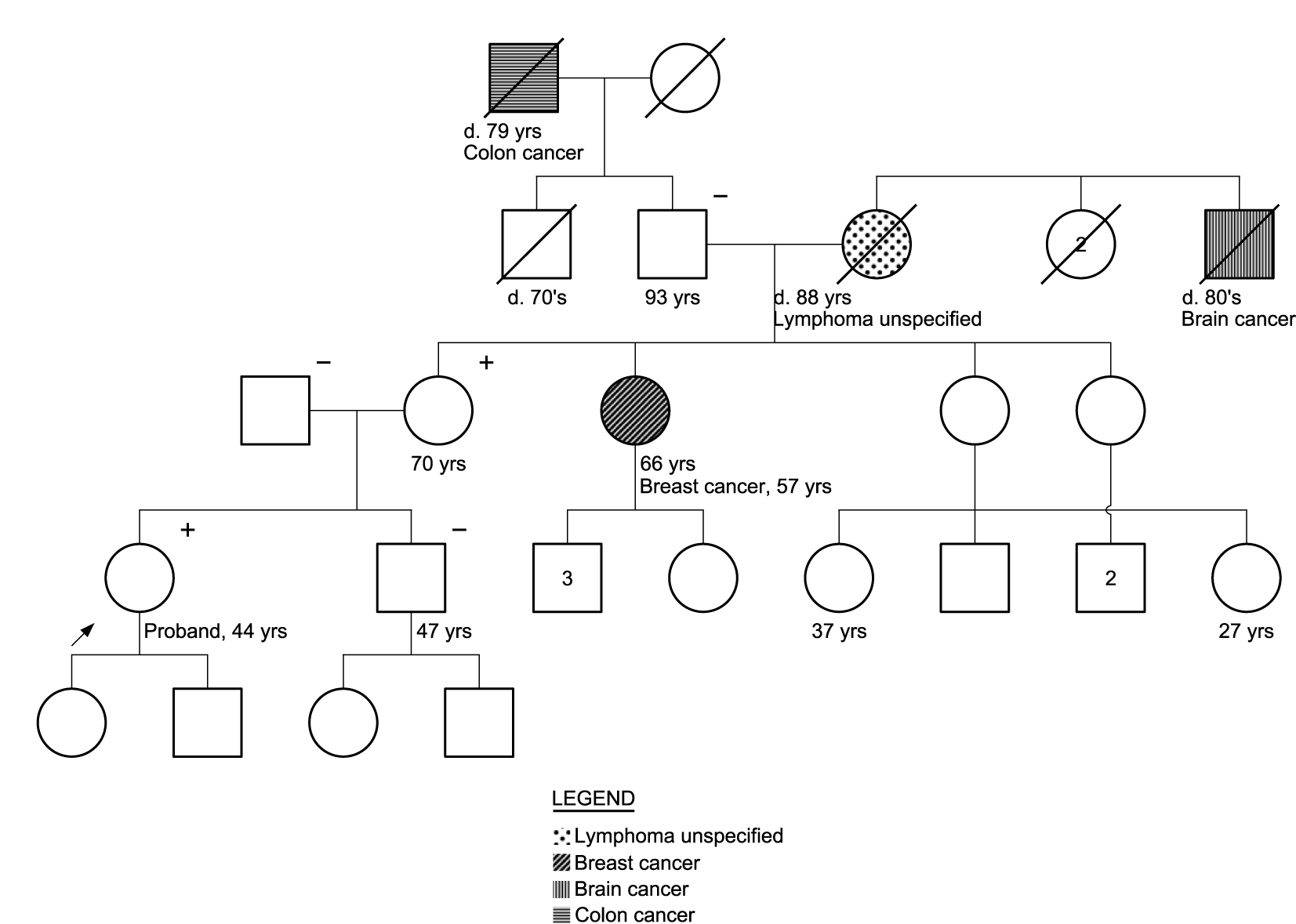

Supplement: Supplementary file 1 — Data S1. Report supporting VUS status of ATM p.S1655Y/c.4964C>A. [file MGG3-4-232-s001.tif]
