# Supplementary material for: Panel testing reveals nonsense and missense CDH1 mutations in families without hereditary diffuse gastric cancer
Source: Mol Genet Genomic Med. 2016 Jan 13;4(2):232–6. doi: 10.1002/mgg3.197 (PMC4799867; doi:10.1002/mgg3.197)

VARIANT DETAILS:

The **p.S1655Y** variant (also known as c.4964C>A), located in coding exon 32 of the *ATM* gene, results from a C to A substitution at nucleotide position 4964. The serine at codon 1655 is replaced by tyrosine, an amino acid with dissimilar properties. This variant was not reported in population based cohorts in the following databases: Database of Single Nucleotide Polymorphisms (dbSNP), NHLBI Exome Sequencing Project (ESP), and 1000 Genomes Project. In the ESP, this variant was not observed in 6499 samples (12998 alleles) with coverage at this position. To date, this alteration has been detected with an allele frequency of approximately 0.004% (greater than 22000 alleles tested) in our clinical cohort. This amino acid position is well conserved in available vertebrate species. In addition, this alteration is predicted to be possibly damaging and deleterious by PolyPhen and SIFT *in silico* analyses, respectively. Since supporting evidence is limited at this time, the clinical significance of p.S1655Y remains unclear.

FAMILY STUDIES PROGRAM:

Ambry Genetics offers complimentary genetic studies for variants of unknown significance (VUSs) meeting specific criteria in appropriate family members. Review of clinical information is required. Additional information, application instructions and required forms, and patient education materials are available at http://ambrygen.com/family-studies-program. For additional information, please email us at GeneticCounselor@ambrygen.com or call 949-900-5500 and ask to speak with a genetic counselor.

Please note that the classification of variants may change over time as additional information becomes available. Alerts are automatically disseminated via fax and/or AmbryPort2 email to clinicians upon variant reclassification. If no updates are received, clinicians are encouraged to contact the laboratory at 949-900-5500 once a year to review the status of previously reported variants.

GENE INFORMATION:

The *ATM* gene, which is classically associated with the autosomal recessive condition ataxia-telangiectasia, is involved in the cellular response to DNA damage and cell-cycle control. Monoallelic pathogenic mutations in this gene have been estimated to confer a 2-4 fold increased risk for female breast cancer compared to the general population; however this risk may be higher for female carriers under the age of 50 (Easton DF. *Int J Radiat Biol.*1994 Dec;66(6 Suppl):S177-82; Thompson D et al. *J Natl Cancer Inst.* 2005 Jun 1;97(11):813-22). In addition, there is evidence that rare missense alterations in ATM, especially those affecting highly-conserved residues in the 3' protein functional domains, act as dominant-negative mutations and are associated with higher breast cancer risks than protein truncating mutations (Gatti R et al. *Mol Genet Metab*. 1999 Dec;68(4):419-23; Tavtigian S et al. *Am J Hum Genet.* 2009 Oct;85(4):427-46; Goldgar D et al. *Breast Cancer Res.* 2011 Jul 25;13(4):R73). Monoallelic mutations in this gene have also been reported in patients with hereditary pancreatic cancer (Roberts NJ et al. *Cancer Discov.* 2012 Jan;2(1):41-6. Epub 2011 Dec 29). Cancer risk estimates for male *ATM* mutation carriers are not currently available. Biallelic pathogenic mutations in the *ATM* gene are known to cause ataxia-telangiectasia (A-T), an autosomal recessive neurodegenerative disorder affecting multiple body systems. Parents who each carry an *ATM* mutation have a 25% chance for a child with A-T in every pregnancy. These risks should be discussed with *ATM* pathogenic mutation carriers of reproductive age.

ADDITIONAL SUPPORTING INFORMATION:

Co-Segregation	Co-segregation data for this variant is currently unavailable.
Co-occurrence	This variant has been detected in conjunction with a pathogenic mutation in *CDH1* by our laboratory (this individual).
Frequency	No population frequency information could be found.
Grantham Score	144 (dissimilar amino acid substitution)
Polyphen	Possibly damaging (0.624)
SIFT	Deleterious (0.040)

ATM NM_000051 c.4964C>A p.S1655Y

Evolutionary conservation diagram: Amino Acid Alignment

This amino acid position is well conserved in available vertebrate species.

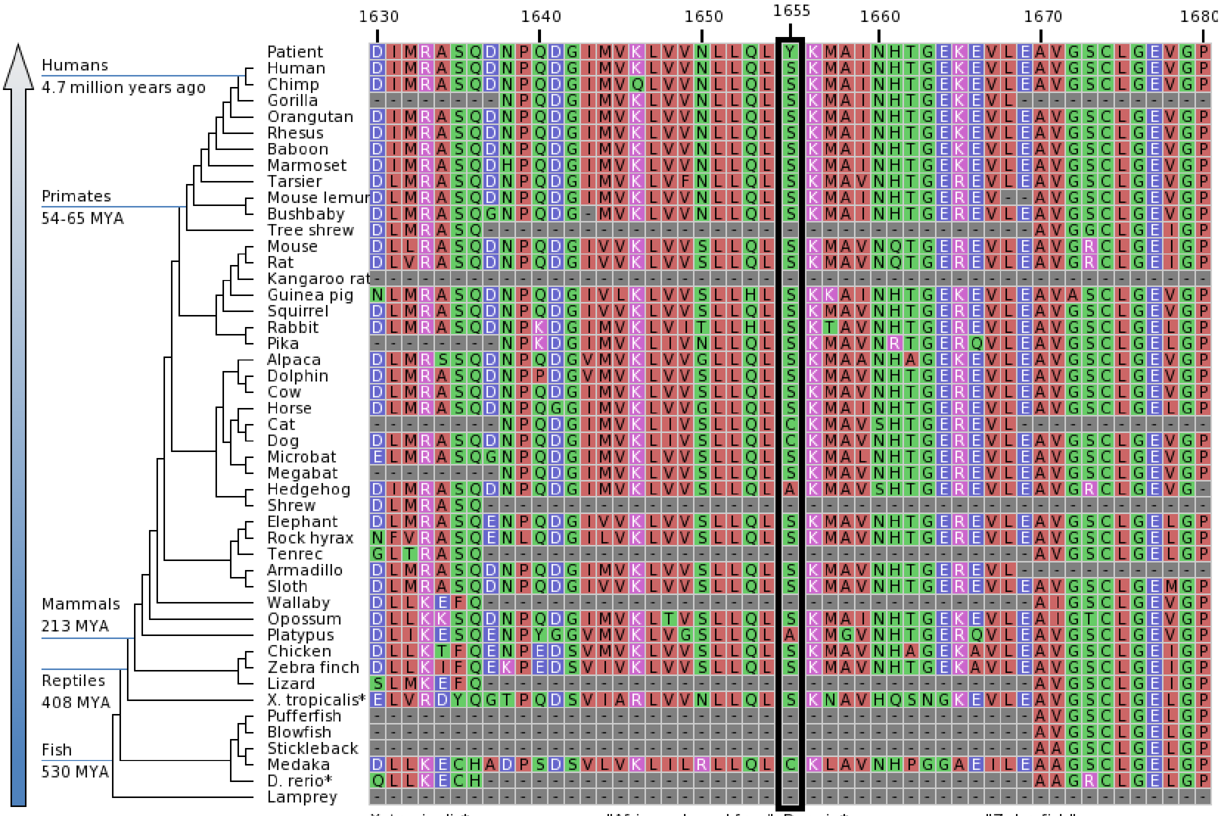

X. tropicalis*: common name "African clawed frog"; D. rerio*: common name "Zebrafish"

Grantham Table:

Amino Acid comparison table		
**Trait**	**Ser (S)**	**Tyr (Y)**
Amino acid name	Serine	Tyrosine
Polarity/Charge	polar	polar
pH	neutral	neutral
Residue weight	87	163
Hydrophobicity score	-0.8	-1.3
Hydrophilicity score	0.3	-2.3
Secondary structure propensity	α indifferent<br>β breaker	α breaker<br>strong β former

Grantham Difference

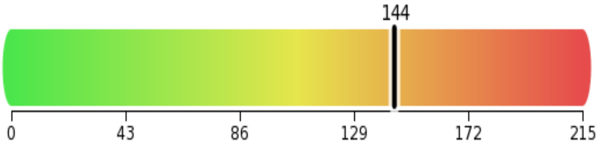

Supplement: Supplementary file 2 — Data S2. Pedigree of family 1. Arrow depicts the proband. Women are represented by circles. Men are represented by squares. A diagonal slash indicates a deceased family member. (+) indicates family members with the ATM p.S1655Y/c.4964C>A variant of unknown significance. (−) indicates tested noncarriers. The legend illustrates cancers affecting the family. [file MGG3-4-232-s002.pdf]
